# Cerebral Hypoperfusion in a sample of patients with Chronic Schizophrenia

**DOI:** 10.1192/j.eurpsy.2026.10512

**Published:** 2026-07-31

**Authors:** A.-P. Taprantzi, G. Georgiou, A. Karampas, M. Plakoutsis, D.-D. Koutsogianni, C. I. Gavrielatos, A. Goudeli, C. Sioka, P. Petrikis

**Affiliations:** 1 Department of Psychiatry; 2 Department of Nuclear Medicine, University of Ioannina, Ioannina, Greece

## Abstract

**Introduction:**

Cerebral hypoperfusion has been found in many neuropsychiatric disorders, it has been attributed to chronic inflammatory responses and may also be linked to the pathophysiology of schizophrenia.

**Objectives:**

The evaluation of brain perfusion abnormalities in a sample of patients with chronic schizophrenia.

**Methods:**

Twenty patients with schizophrenia were included in the study. 90% were males, aged 27-68 years with a 1-10 number of hospitalizations and 2-38 years duration of illness. All of them received antipsychotic medications. Patients with a history of somatic disorders or/and substance abuse were excluded from the study. All participants gave informed consent. They underwent a 99mTc-HMPAO-SPECT RCP. The assessment of the brain’s regional cerebral blood flow (rCBF) was performed using an automated NeurogamTM scanning data analysis program. Abnormal perfusion areas were considered those below two standard deviations from the normal mean uptake in area> 50% pixels.

**Results:**

Compared with demographically adjusted normative data, patients with schizophrenia presented significant hypoperfusion bilaterally in frontal, limbic and temporal regions. More specifically, reduced r CBF was most extensive in the limbic lobe bilaterally in the vast majority of the study group (16/20, 80% left, 17/20, 85% right), in the temporal lobe (7/20 left, 2/20 right), frontal lobe (3/ 20 left, 1/20 right), parietal lobe (3/20 left, 1/20 right), and occipital lobe (one and two out of 20 respectively).

Most patients (N=17, 85%) showed abnormal hypoperfusion in BA 28 (ventral entorhinal cortex), bilaterally, 16 patients (80% of the sample) in BA 38 (temporopolar area), bilaterally, 15 patients in BA 36 (perirhinal cortex) bilaterally, while 12 and 14 patients showed hypoperfusion in BA 25 (subgenual area, part of the ventromedial prefrontal cortex) left and right respectively while half of the study group presented reduced r CBF in BA24 (anterior cingulated cortex), bilaterally. Half of the patients showed hypoperfusion in BA20 (inferior temporal gyrus) left and six in BA20 right. Seven and six patients, respectively, showed hypoperfusion in BAs 23 (posterior cingulate cortex) and 11 (orbitofrontal area), bilaterally.

**Conclusions:**

Patients with schizophrenia show extensive hypoperfusion, mostly in the limbic lobes, bilaterally. The most affected BAs were BA 28,38,36,25 and 24, bilaterally.

**Disclosure of Interest:**

None Declared

